# Cerebrospinal fluid levels of inflammation, oxidative stress and NAD^+^ are linked to differences in plasma carotenoid concentrations

**DOI:** 10.1186/1742-2094-11-117

**Published:** 2014-07-01

**Authors:** Jade Guest, Ross Grant, Manohar Garg, Trevor A Mori, Kevin D Croft, Ayse Bilgin

**Affiliations:** 1 Australasian Research Institute, Sydney Adventist Hospital, Sydney, NSW, Australia; 2 School of Medical Sciences, Faculty of Medicine, University of New South Wales, Wallace Wurth Building, office #203, Sydney, NSW 2052, Australia; 3 Sydney Medical School, University of Sydney, Sydney, NSW, Australia; 4 School of Biomedical Sciences and Pharmacy, University of Newcastle, Newcastle, NSW, Australia; 5 School of Medicine and Pharmacology, Royal Perth Hospital Unit, University of Western Australia, Perth, WA, Australia; 6 Faculty of Science, Macquarie University, Sydney, NSW, Australia

**Keywords:** Brain, Carotenoid, Inflammation, NAD^+^, Oxidative stress

## Abstract

**Background:**

The consumption of foods rich in carotenoids that possess significant antioxidant and inflammatory modulating properties has been linked to reduced risk of neuropathology. The objective of this study was to evaluate the relationship between plasma carotenoid concentrations and plasma and cerebrospinal fluid (CSF) markers of inflammation, oxidative stress and nicotinamide adenine dinucleotide (NAD^+^) in an essentially healthy human cohort.

**Methods:**

Thirty-eight matched CSF and plasma samples were collected from consenting participants who required a spinal tap for the administration of anaesthetic. Plasma concentrations of carotenoids and both plasma and cerebrospinal fluid (CSF) levels of NAD(H) and markers of inflammation (IL-6, TNF-α) and oxidative stress (F2-isoprostanes, 8-OHdG and total antioxidant capacity) were quantified.

**Results:**

The average age of participants was 53 years (SD = 20, interquartile range = 38). Both α-carotene (*P* = 0.01) and β-carotene (*P* < 0.001) correlated positively with plasma total antioxidant capacity. A positive correlation was observed between α-carotene and CSF TNF-α levels (*P* = 0.02). β-cryptoxanthin (*P* = 0.04) and lycopene (*P* = 0.02) inversely correlated with CSF and plasma IL-6 respectively. A positive correlation was also observed between lycopene and both plasma (*P* < 0.001) and CSF (*P* < 0.01) [NAD(H)]. Surprisingly no statistically significant associations were found between the most abundant carotenoids, lutein and zeaxanthin and either plasma or CSF markers of oxidative stress.

**Conclusion:**

Together these findings suggest that consumption of carotenoids may modulate inflammation and enhance antioxidant defences within both the central nervous system (CNS) and systemic circulation. Increased levels of lycopene also appear to moderate decline in the essential pyridine nucleotide [NAD(H)] in both the plasma and the CSF.

## Background

Neuroinflammation and oxidative stress have emerged as key players in the complex interplay between environmental and biological factors involved in the development of neurodegenerative disease [1,2]. Under normal homeostatic conditions both immune and oxidative activity are largely transitory due to inherent negative feedback mechanisms including increased production of anti-inflammatory cytokines and enhanced endogenous antioxidant (that is glutathione peroxidase, catalase or superoxide dismutase) activity. During periods of chronic disease however, these processes can be continuously activated often amplifying each other in a positive feed forward cycle, causing cell death, tissue dysfunction and disease [3,4].

Neuroinflammation is a well-established feature of the pathology associated with most neurodegenerative disorders [5-7]. During various stages of disease the activity of microglia and astrocytes are markedly increased resulting in altered production of important signalling molecules such as cytokines [8,9]. Secretion of the inflammatory cytokines TNF and IL-6 are reportedly up-regulated within the central nervous system (CNS) of a number of neurodegenerative disorders including Alzheimer’s disease (AD) [10]. While elevated levels of TNF and IL-6 have been found to induce neuronal apoptosis and tau protein phosphorylation respectively [11-14], observations that IL-6 and TNF inhibit glutamate and β-amyloid (Aβ) toxicity respectively also indicate potentially neuroprotective roles [15,16]. At lower concentrations TNF has also been shown to be involved in synaptic scaling, cell signalling and a number of behavioural and autonomic processes, leading some to consider this cytokine as a neuromodulator [17-22]. Likewise, physiological levels of IL-6 have been shown to possess neuromodulatory properties, enhancing the differentiation of neurons and expression and function of the neuronal adenosine A1 receptor, an important modulator of synaptic transmission [23,24]. Evidence is thus emerging that cytokines such as TNF and IL-6 can elicit either positive modulatory or toxic effects in the CNS that is likely dependent on their level of expression.

While the complex pathological and physiological roles of inflammatory cytokines in the CNS are yet to be fully understood, it is relatively well established that elevated levels of each of these cytokines directly promote oxidative stress [25-27]. The brain, and neurons in particular, are especially vulnerable to oxidative stress as a consequence of their poor antioxidant protection, high oxygen demand and high levels of both polyunsaturated fatty acids and transition metals [28]. The term ‘oxidative stress’, describes a significant imbalance between antioxidant defences and the bodies’ formation of reactive nitrogen (RNS) and/or oxygen species (ROS) [29]. Accumulation of RNS/ROS may result in a variety of detrimental effects such as protein oxidation, lipid peroxidation and DNA damage [2]. Failure to repair this damage, particularly to the DNA, has been demonstrated to cause genomic instability and neuronal apoptosis and is associated with the development of several neuropathologies [2,30-32].

In order to maintain cellular integrity and homeostasis after oxidative assault, a number of repair processes exist including activation of the nicotinamide adenine dinucleotide (NAD^+^) dependent DNA nick sensor poly(ADP-ribose) polymerase-1 (PARP) [33]. Importantly PARP activation in response to DNA damage catalyses the successive cleavage of the ADP-ribose moiety from NAD^+^ resulting in the formation of poly(ADP-ribose) subunits. In the context of low-to-moderate DNA damage this process facilitates DNA repair. However, over-activation of PARP, due to excessive DNA damage, can result in neuronal death as a consequence of decreased ATP production following critical depletion of NAD^+^[34]. In order to preserve cellular energy and concomitantly PARP activity, adequate levels of NAD^+^ must be maintained.

While age is the major risk factor for the development of most neurodegenerative disorders [35,36], a number of lifestyle choices have been linked to either promotion or prevention of pathogenesis by increasing or decreasing oxidative stress and inflammation. Carotenoids, a family of phytochemicals synthesised by plants, that are responsible for the red, orange, and yellow pigments of fruit and vegetables, have been the subject of increased attention as a result of their antioxidant and inflammatory modulating properties. Importantly, serum carotenoids have been shown to be positively associated with brain carotenoid levels and can be considered a reliable predictor of brain carotenoid concentrations [37].

Within the brain, carotenoids are thought to exert a variety of protective effects. In a cell culture model of AD, lycopene was shown to efficiently attenuate Aβ-induced ROS formation, improve neuron viability and decrease the rate of apoptosis [38]. Low serum levels of lycopene, β-carotene, lutein and zeaxanthin have also been linked to impaired cognitive function and AD [39-42]. Lutein supplementation has also been shown to prevent erythrocyte lipid peroxidation in humans and has been suggested as a potential therapy for the prevention of dementia [43].

Collectively, these reports indicate that higher carotenoid levels may mitigate against the development of an oxidative-inflammatory state and thereby reduce tissue damage within the CNS. While evidence from cell culture, animal and limited *post-mortem* brain tissue studies support this hypothesis, to date no study has investigated this putative association in the CNS of a healthy human cohort. Therefore the objective of this study was to evaluate the relationship between plasma concentrations of carotenoids and plasma and cerebrospinal fluid (CSF) markers of inflammation, oxidative stress and NAD^+^ in an essentially healthy human cohort.

## Methods

### Ethics statement

This study was conducted in accordance with the Helsinki declaration. Ethical approval was obtained from the Human Research Ethics Committee, Sydney Adventist Hospital (EC00141, project number 2011-005). Written informed consent was obtained from all participants prior to commencement.

### Participants

Male (n = 10) and female (n = 28) participants, who required a spinal tap for the administration of anaesthetic as part of routine care (that is prior to surgery), were recruited at Sydney Adventist Hospital, Australia as part of a larger health study [44]. The average age of participants was 53 years (SD = 20, interquartile range = 38). Participants were excluded from the cohort if they were smokers or had a confirmed diagnosis of a neurological/neurodegenerative disorder or CNS infection. In total thirty-eight CSF and matched blood samples were collected from consenting participants considered in general good health.

### Sample collection

CSF and blood samples (fasting ≥ ten hours) were collected by an anaesthetist no longer than 30 minutes apart. Blood was collected into heparinised tubes from a superficial vein on an upper limb, prior to the administration of fluids or anaesthetics. CSF samples were collected via standard lumber puncture prior to injection of anaesthetics. Samples were centrifuged for 10 minutes at 1,800 rpm and stored at −194°C within 1 hour of collection, for a maximum of 12 months, until analysis. Samples intended for F2-isoprostane analysis where stored in the presence of a glutathione/butylated hydroxytoluene preservative.

### Biochemical analysis

Total nicotinamide adenine dinucleotide (NAD(H)) concentrations were measured spectrophotometrically using a thiazolyl blue microcycling assay established by Bernofsky and Swan [45], and adapted to a 96-well plate format by Grant and Kapoor as previously described [46,47].

IL-6, TNF-α, total antioxidant capacity (TAC) and 8-hydroxy-2′-deoxyguanosine (8-OHdG) were measured using a standardised commercial solid phase sandwich enzyme-linked immunosorbent assay (ELISA) (Human IL6 High Sensitivity ELISA Kit, Abcam, Cambridge, MA, USA; Human TNF-α UltraSensitive, Invitrogen Corporation, Camarillo, CA, USA; Antioxidant Assay Kit, Cayman Chemical Company, Ann Arbor, MI, USA; Highly Sensitive 8-OHdG Check, Japan Institute for the Control of Ageing, Shizuoka Japan).

Total F2-isoprostanes were measured by gas chromatography-mass spectrometry (GC-MS) using electron capture negative ionisation according to a modification in the method of Mori *et al*. as previously described [48,49].

**Figure 1:**
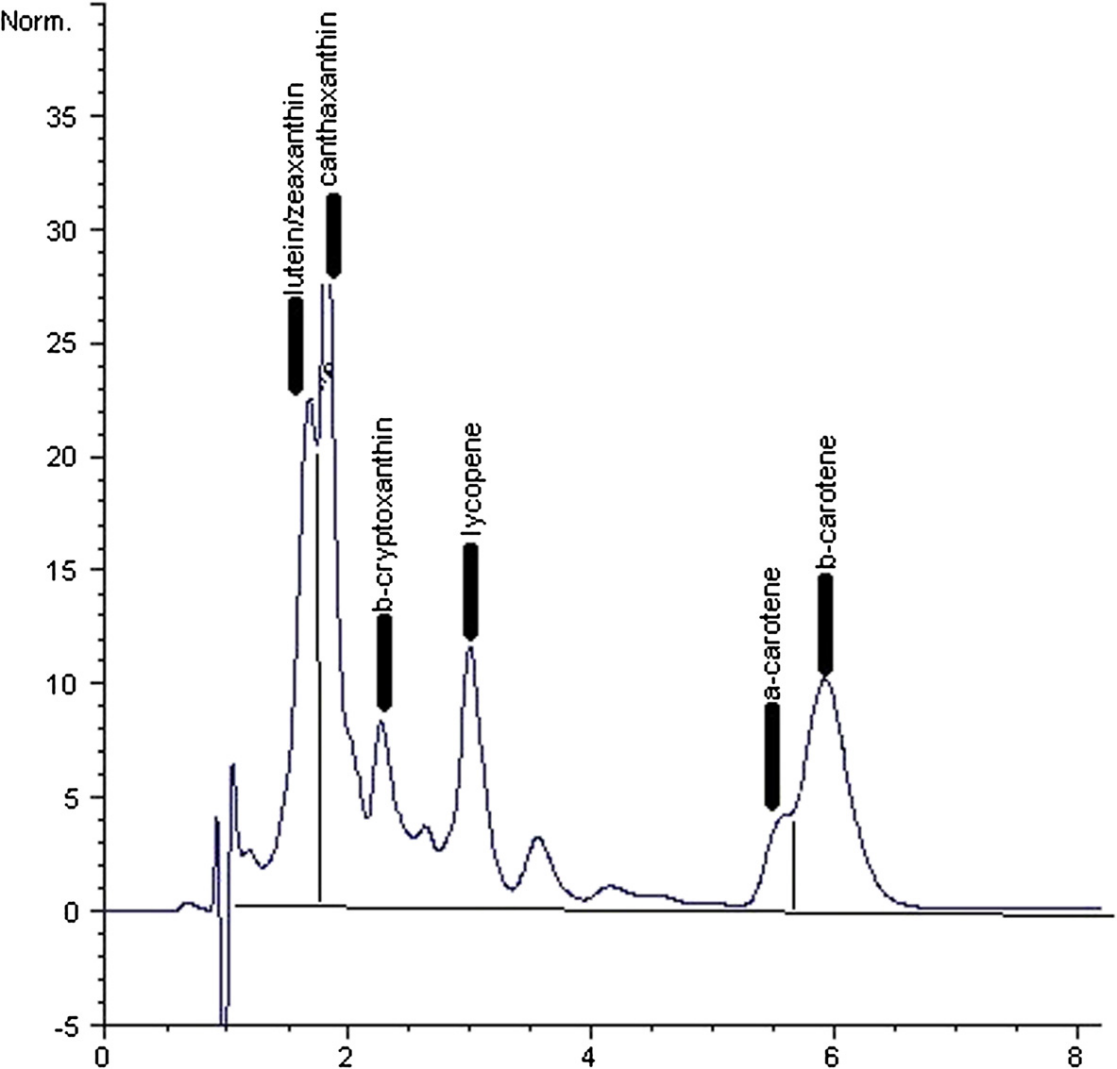
An example chromatograph of plasma carotenoids as measured by HPLC.

Plasma carotenoids were measured using high-performance liquid chromatography according to the method established by Barua and colleagues and previously described (Figure 1) [50,51].

### Statistical analysis

Statistical analyses were performed using SPSS version 16.0 for Windows (SPSS Inc., Chicago, IL, USA). Data is presented as means ± standard deviation (SD) unless otherwise stated. The Pearson correlation coefficient and multiple linear regression, controlling for age and gender, was used to identify significant relationships between carotenoids, [NAD(H)], and markers of oxidative stress and inflammation. Normality was assessed using histogram, Shapiro-Wilk and Kolmogorov-Smirnov analysis. Because some carotenoids (lutein and zeaxanthin, β-cryptoxanthin, lycopene, total carotenoids) and both plasma and CSF IL-6 showed slightly skewed distributions, analyses were performed using base-10 log-transformed data. Adjusted and non-adjusted *P*-values are provided throughout with test significance set at *P*-value ≤ 0.05.

## Results

The mean plasma concentrations of individual carotenoid pigments are presented in Table 1.

**Table 1:** Mean plasma carotenoid concentrations

**Carotenoid**	**Mean (ng/mL) ± SD**
Lutein and zeaxanthin	538.24 ± 338.15
β-cryptoxanthin	59.54 ± 54.91
Lycopene	218.86 ± 176.80
α-carotene	15.19 ± 14.92
β-carotene	80.46 ± 82.13
Total carotenoids	935.02 ± 474.95

### Association between carotenoids and inflammatory cytokines

**Figure 2:**
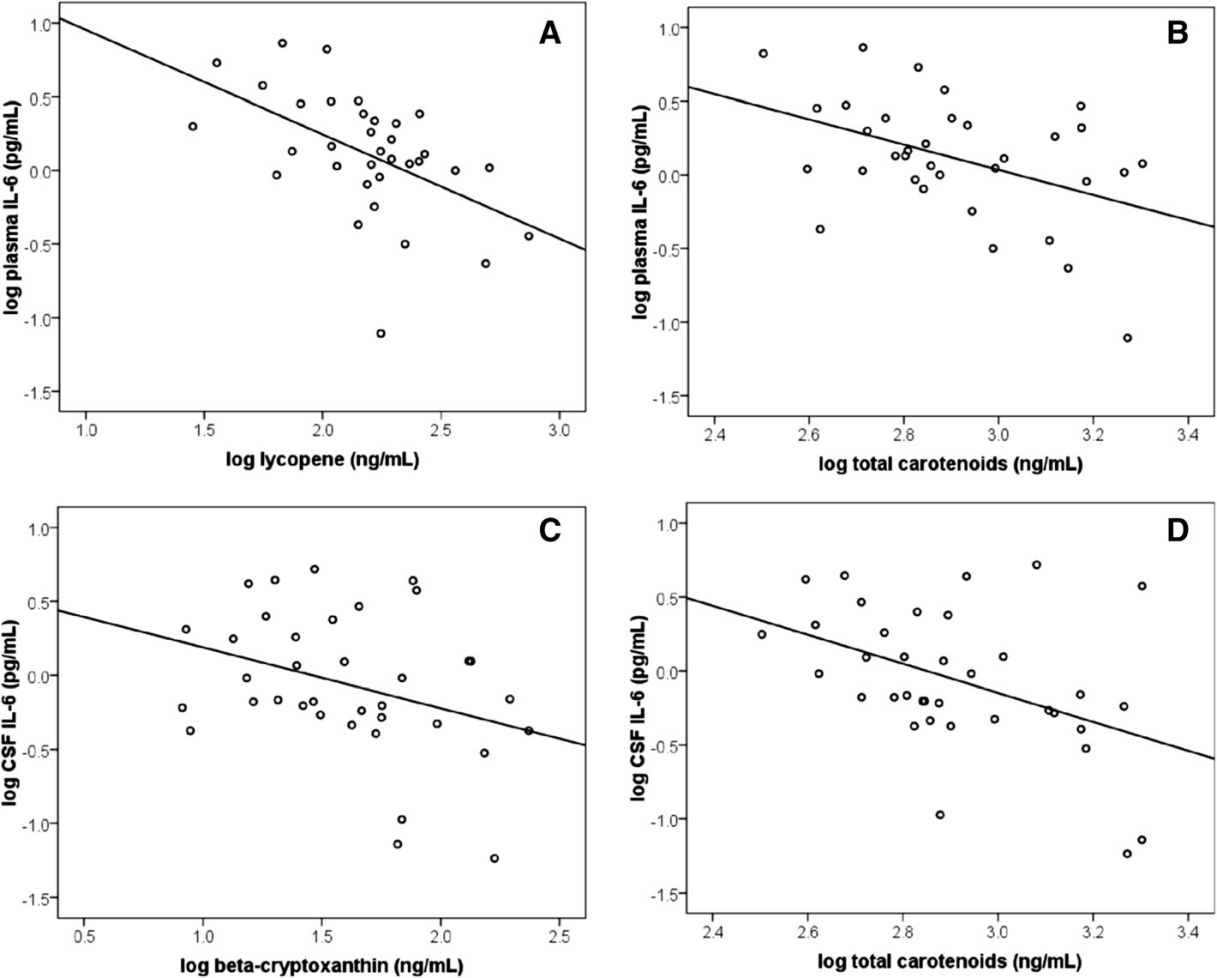
**Association between carotenoids and reduced levels of peripheral and central IL-6. (A) Inverse association between plasma lycopene and plasma IL-6 levels.** A significant inverse association exists between plasma lycopene and plasma IL-6 (*P* = 0.02, R^2^ = 0.21, n = 34). Comparisons were made using multiple linear regression controlling for age and gender. **(B)** Inverse association between plasma total carotenoids and plasma IL-6 levels. A significant inverse association exists between total plasma carotenoids and plasma IL-6 (*P* = 0.01, r = −0.44, n = 34), however this relationship does not remain statistically significant after controlling for age and gender (*P* = 0.06). Comparisons were made using the Pearson correlation coefficient and multiple linear regression controlling for age and gender. **(C)** Inverse association between plasma β-cryptoxanthin and cerebrospinal fluid (CSF) IL-6 levels. A significant inverse association exists between plasma β-cryptoxanthin and CSF levels of IL-6 (*P* = 0.04, R^2^ = 0.45, n = 36). Comparisons were made using multiple linear regression controlling for age and gender. **(D)** Inverse association between total carotenoids and CSF IL-6 levels. A significant inverse association exists between plasma total carotenoids and CSF levels of IL-6 (*P* = 0.01, r = −0.44, n = 36), however this relationship does not remain statistically significant after controlling for age and gender (*P* = 0.06). Comparisons were made using the Pearson correlation coefficient and multiple linear regression controlling for age and gender.

A significant inverse association was observed between plasma levels of lycopene and plasma IL-6 (*P* < 0.001, r = −0.53, n = 34) (Figure 2A). This association remained even after controlling for age and gender (*P* = 0.02, R^2^ = 0.21). An increase in lycopene of 1% was associated, on average, with a 0.54% decrease in plasma IL-6. Higher plasma total carotenoid concentrations were also associated with lower levels of plasma IL-6 (*P* = 0.01, r = −0.44, n = 34) (Figure 2B), however this relationship did not remain statistically significant after controlling for age and gender (*P* = 0.06). No associations were found between plasma IL-6 and any other individual carotenoid.

An increase in plasma β-cryptoxanthin levels was associated with a decrease in CSF IL-6 (*P* = 0.04, r = −0.34, n = 36) (Figure 2C). This association remained even after controlling for age and gender (*P* = 0.04, R^2^ = 0.45). An increase in β-cryptoxanthin by 1% was associated, on average, with a 0.33% decrease in CSF IL-6. Higher plasma total carotenoids were also associated with reduced levels of CSF IL-6 (*P* = 0.01, r = −0.44, n = 36) however this relationship did not remain statistically significant after controlling for age and gender (*P* = 0.06) (Figure 2D). A trend between increased plasma lycopene levels and reduced concentrations of CSF IL-6 was observed, however this was not statistically significant (*P* = 0.22). No associations were found between CSF IL-6 and any other individual carotenoid.

**Figure 3:**
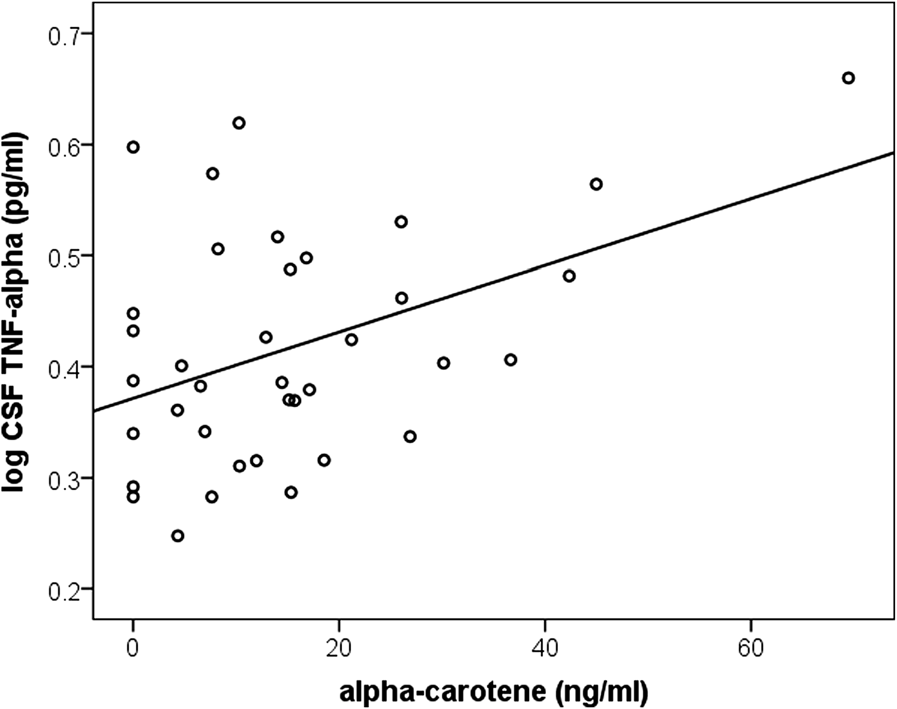
**Positive association between cerebrospinal fluid (CSF) TNF-α and plasma α-carotene.** A significant positive association exists between plasma α-carotene and CSF TNF-α (*P* = 0.02, R^2^ = 0.10, n = 36). Comparisons were made using multiple linear regression controlling for age and gender.

An increase in plasma α-carotene was associated with an increase in CSF TNF-α (*P* = 0.01, r = 0.185, n = 36) (Figure 3). This association remained even after controlling for age and gender (*P* = 0.02, R^2^ = 0.10). A ten-fold increase in α-carotene was associated with a small 0.003 pg/mL increase in CSF TNF-α. A positive trend between β-carotene and CSF TNF-α was also observed, however this did not reach statistical significance (*P* = 0.17). No associations were found between CSF TNF-α and any other individual carotenoid.

### Association between carotenoids and markers of oxidative stress

**Figure 4:**
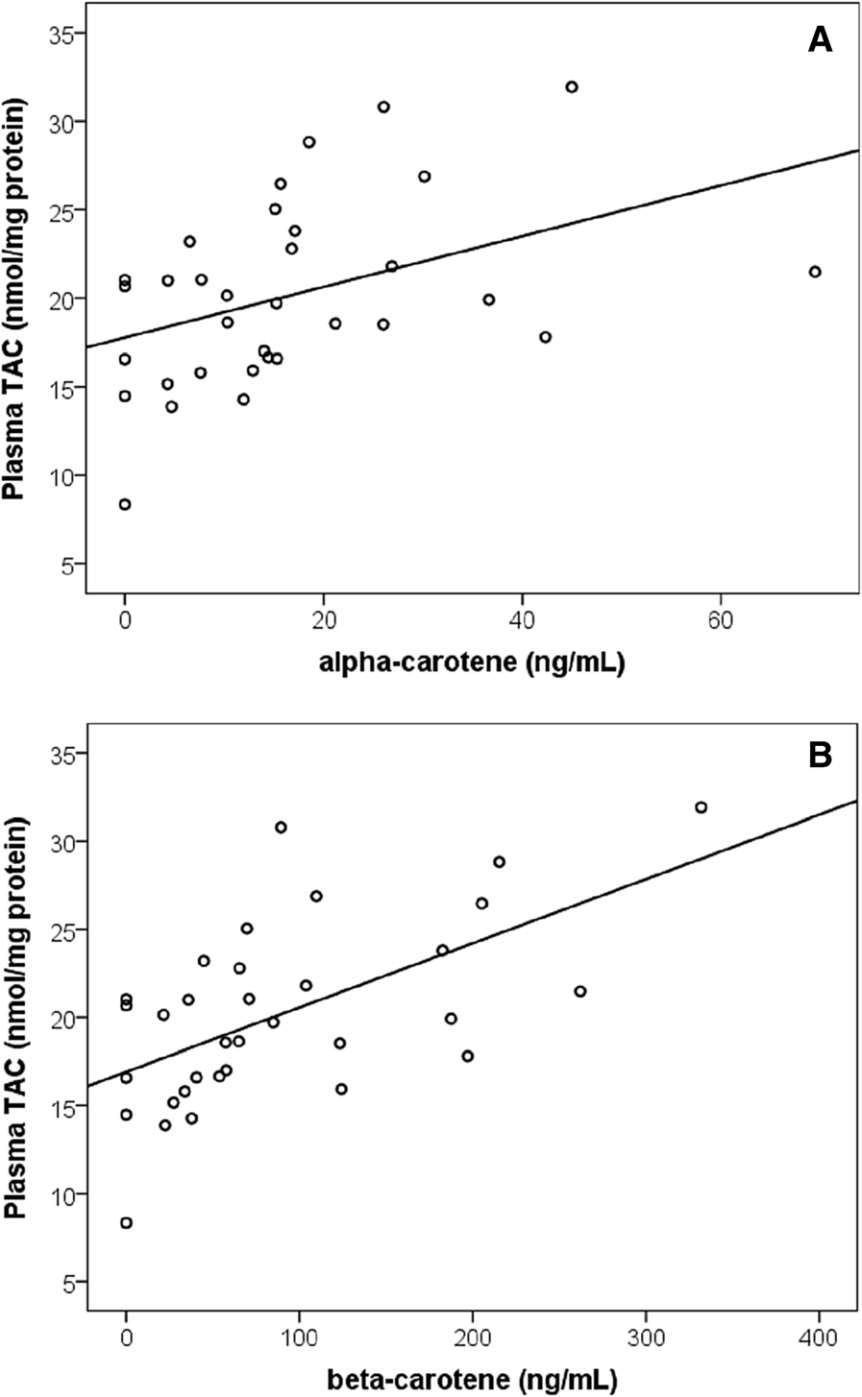
**Positive association between plasma total antioxidant capacity (TAC) and (A) plasma α-carotene (B) plasma β-carotene. (A)** A significant positive association exists between plasma α-carotene and plasma TAC (*P* = 0.01, R^2^ = 0.14, n = 33). Comparisons were made using multiple linear regression controlling for age and gender. **(B)** A significant positive association exists between plasma β-carotene and plasma TAC (*P* < 0.001, R^2^ = 0.32, n = 33). Comparisons were made using multiple linear regression controlling for age and gender.

A significant positive association was observed between both α-carotene (*P* = 0.01, r = 0.43, n = 33) and β-carotene (*P* < 0.001, r = 0.59, n = 33) and the total antioxidant capacity (TAC) in plasma (Figure 4). These relationships remained even after controlling for age and gender (*P* = 0.01, R^2^ = 0.14; *P* < 0.001, R^2^ = 0.32 respectively). A 1 ng/mL increase in α-carotene or β-carotene was associated with a 0.15 or 0.04 nmol/mg protein increase in plasma TAC respectively. When controlling for age and gender, higher plasma levels of lycopene also tended to correspond with reduced levels of plasma F2-isoprostanes, however this did not quite reach statistical significance (*P* = 0.06). An apparent inverse trend between CSF F2-isoprostane levels and plasma β-cryptoxanthin (*P* = 0.16), α-carotene (*P* = 0.14) and β-carotene (*P* = 0.20) was observed, though these did not reach statistical significance. No further associations were apparent between CSF F2-isoprostane and plasma levels of any individual carotenoid. No associations were observed between CSF 8-OHdG concentrations and plasma levels of any individual carotenoid.

### Association between carotenoids and both peripheral and central [NAD(H)]

**Figure 5:**
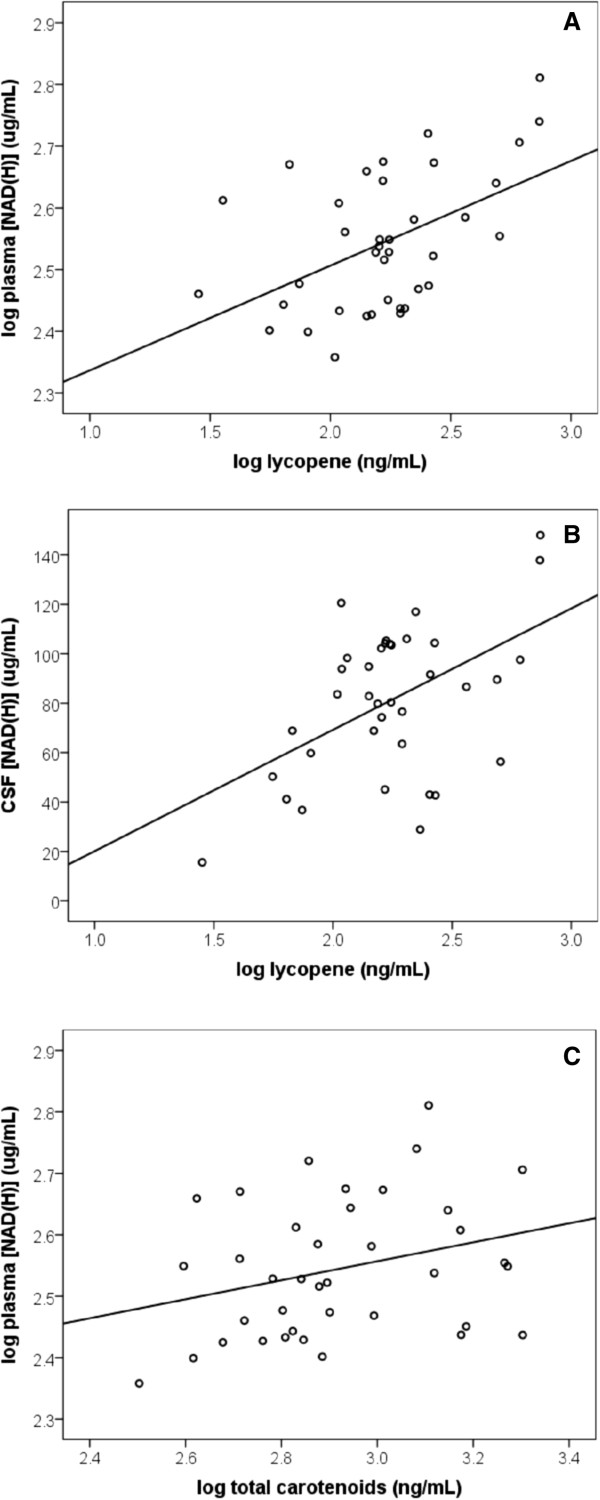
**Association between carotenoids and increased levels of peripheral and central [NAD(H)]. (A)** A significant positive association exists between lycopene and plasma [NAD(H)] (*P* < 0.001, R^2^ = 0.31, n = 38). Comparisons were made using multiple linear regression controlling for age and gender. **(B)** A significant positive association exists between lycopene and CSF [NAD(H)] (*P* < 0.001, R^2^ = 0.21, n = 37). Comparisons were made using multiple linear regression controlling for age and gender. **(C)** A significant positive association exists between total carotenoids and plasma [NAD(H)] (*P* = 0.03, R^2^ = 0.12, n = 37). Comparisons were made using multiple linear regression controlling for age and gender.

A significant positive association was found between lycopene and both plasma (*P* < 0.01, r = 0.50, n = 38) and CSF (*P* < 0.01, r = 0.50, n = 37) [NAD(H)] (Figure 5). These relationships remained even after controlling for age and gender (*P* < 0.001, R^2^ = 0.31; *P* < 0.001, R^2^ = 0.21 respectively). A 1% increase in lycopene was associated, on average, with a 0.20% increase in plasma [NAD(H)]. Likewise a ten-fold increase in lycopene was associated with a 48.4 μg/mL increase in CSF [NAD(H)].

After controlling for age and gender a positive association between total carotenoids and plasma NAD(H) levels was also observed (*P* = 0.03, R^2^ = 0.12, n = 37) (Figure 5C). A 1% increase in total carotenoids was associated, on average, with a 0.21% increase in plasma [NAD(H)]. Plasma total carotenoids were positively associated with CSF [NAD(H)] (*P* = 0.05, r = 0.33, n = 37), however this did not remain statistically significant after controlling for age and gender. No associations were found between plasma [NAD(H)], CSF [NAD(H)] and any other individual carotenoid.

## Discussion

Neuroinflammation and oxidative stress are established pathological features of most neurodegenerative disorders. While the consumption of foods rich in carotenoids, known to possess antioxidant and inflammatory modulating properties, has been linked to a reduced risk of cognitive decline [40], surprisingly there is limited research on the effect of carotenoids on neuroinflammation and oxidative stress. To our knowledge this is the first study to investigate the association between peripheral carotenoid concentrations and CNS markers of oxidative stress and inflammation in humans without CNS pathology.

As expected, high plasma lycopene and total carotenoid concentrations were associated with reduced plasma IL-6 levels. This is consistent with epidemiologic studies reporting carotenoid concentrations to be inversely associated with peripheral markers of inflammation [52]. While further research into the mechanism of carotenoid mediated inflammation is required, it has been demonstrated that a variety of carotenoids, including lycopene and lutein inhibit cytokine production via suppressing ROS stimulated NF-κβ activation [53-56].

While major contributors to plasma TAC are uric acid and albumin [57], the positive correlation observed between plasma TAC and both α-carotene and β-carotene indicate that these carotenoids contribute to peripheral antioxidant defences. However, their capacity to mitigate against both lipid and DNA damage *in vivo* is questionable as no inverse associations were observed between carotenes and either plasma F2-isoprostane or 8-OHdG respectively. Previous evidence for an association between the carotenes and markers of lipid and DNA oxidative damage is mixed, with some studies showing a significant protective effect and others finding no significant association [58-61]. For example van den Berg and colleagues reported that while a three-week plant-based diet increased serum levels of carotenoids, including α-carotene and β-carotene, and increased TAC, consistent with results from this study, no effects were observed on any plasma marker of oxidative damage to lipids, proteins and DNA [61].

A number of carotenoids, including β-cryptoxanthin, lycopene and β-carotene, are thought to cross the blood brain barrier and modulate inflammation [62,37]. In this study a novel inverse association between β-cryptoxanthin and CSF IL-6 was observed. Similarly an increase in total carotenoids was also associated with reduced CSF IL-6 levels. This is consistent with *in vitro* work by others showing that β-cryptoxanthin significantly attenuates both the release and transcription of various cytokines [63,64]. Astaxanthin, another member of the carotenoid xanthophyll family, has also been shown to decrease IL-6 mRNA in activated microglia [65].

In contrast to the inverse associations observed between carotenoids and IL-6, an increase in plasma α-carotene was associated with a statistically significant increase in CSF TNF-α. A positive, though not statistically significant trend was also observed between β-carotene and CSF TNF-α. While evidence of the moderating effect of carotenoids on inflammation is growing, consistent with results from this study, a small number of *in vitro* and *ex vivo* studies have reported that β-carotene can increase the secretion of TNF-α from monocytes and macrophages [66,67]. Although no known studies have investigated the potential for α-carotene to likewise promote TNF-α secretion, the structural similarity between both carotene types suggest this possibility and is consistent with the data presented in this manuscript, though further research is required to verify this observation.

TNF-α is a multifunctional, pleiotropic cytokine initially identified as a potent regulator of cellular activity during an immune response. Although traditionally considered toxic and known to initiate neuronal apoptosis when elevated [13] in healthy brain tissue under non-inflammatory conditions, physiological TNF-α levels below approximately 20 (pg/mL) appear to have positive neuromodulatory activity [17,68-72]; being involved in synaptic scaling, cell signalling and a number of behavioural and autonomic processes [17-21]. Under certain circumstances TNF-α has also displayed neuroprotective properties such as preventing Aβ toxicity [15]. While the role of TNF-α in normal brain function is yet to be fully elucidated, considered together, these studies indicate that physiological levels of TNF-α, as observed in this study (2.69 pg/mL ± 0.68 SD) may play a role in maintaining neural homeostasis. Therefore, our finding of a positive association between the carotenes and TNF-α levels (within the lower physiological range) likely do not reflect increased inflammation, but rather promotion of the healthy neuromodulatory function of TNF-α.

There is a growing interest in understanding the role and mechanism of the carotenoids as inhibitors of oxidative stress. Indeed evidence suggests that the ability of carotenoids to combat oxidative stress does not solely rely on their capacity to quench free radicals. Recently lycopene, a potent singlet oxygen (^1^O_2_) quencher [73], has been reported to restore PARP activity after deltamethrin-induced testicular injury in rats [74]. Further Apo-10′ lycopenoic acid, a lycopene metabolite, has been shown in murine models to increase sirtuin 1 (SIRT1) mRNA and protein levels [75]. Importantly, both PARP and SIRT1 use NAD^+^ as the primary substrate for their activities. We report for the first time a significant positive association between lycopene and both plasma and CSF NAD(H) levels, which remained even after controlling for age and gender. While further investigation is required, we postulate that by quenching ROS, lycopene reduces DNA damage, preventing PARP over-activation, and hence preservation of central NAD^+^ stores.

NAD^+^ is a ubiquitous molecule that is necessary for a number of vital cellular processes. In addition to its role in classical energy production and many cellular reactions as a redox couple, NAD^+^ also serves, as previously mentioned, as a substrate for the sirtuin and PARP family of enzymes and the immune linked NAD^+^ glycohydrolases (CD38) [76-78]. As a group, these NAD^+^ dependent enzymes help modulate circadian rhythms, insulin secretion, genomic stability and cell longevity [79-83]. As NAD(H) levels impact at least PARP and SIRT1 activity, our observation that lycopene correlates with increased NAD(H) availability within the brain suggests that consumption of this carotenoid may improve cell metabolic and genomic stability, decreasing an individuals’ susceptibility to neurodegenerative disease. This is of potential significant relevance in light of observations by others that higher levels of NAD+ correlate with reduced synaptic loss and increased neuronal viability [84,85], and the need to find effective strategies to maintain cell function in the aging brain.

While the observations reported in this study are statistically valid, it is recognised that these associations have been obtained from a modest number of matched whole blood and CSF samples, potentially limiting sensitivity. As a result, some relationships may have been obscured; for example, although no statistically significant association between carotenoids and specific biomarkers of lipid and DNA damage was found, a number of expected inverse trends were observed that may have reached statistical significance if sample numbers were increased. In addition, some observed associations did not remain significant after controlling for age and gender (for example, between CSF IL-6 and serum total carotenoids) which may yet prove significant in a larger cohort study. In the case of age, this may also reflect the relative inability of carotenoids to exert enough anti-inflammatory effect on their own to combat the known age associated acceleration in inflammation and oxidative stress [47,86]. Nevertheless the reported associations in this study are generally consistent with the limited number of previously published observations and represent the first data linking carotenoid levels with changes in [NAD(H)] and inflammatory/oxidative stress markers in the healthy CNS.

## Conclusion

An extensive body of evidence now indicates that oxidative stress and inflammation play a central role in the development of neurodegenerative disorders. While the consumption of foods rich in carotenoids have been linked to a reduced risk of this type of neuropathology, limited research is available detailing their association with inflammation and oxidative stress levels within the human CNS. This study provides evidence for the first time that plasma carotenoids may modulate CSF levels of inflammatory cytokines in healthy humans and reports a link between increased plasma carotenoid concentrations and reduced oxidative activity. We also provide novel evidence that lycopene may influence levels of the essential pyridine nucleotide NAD(H) in both the plasma and the CSF. This data therefore suggests that higher carotenoid intake may assist in the maintenance of brain health, however further research (both cross-sectional and longitudinal) is required to confirm this proposition.

## Abbreviations

8-OHdG: 8-hydroxy-2′-deoxyguanosine; Aβ: beta amyloid; AD: Alzheimer’s disease; ATP: adenosine triphosphate; CD38: cluster of differentiation; CNS: central nervous system; CSF: cerebrospinal fluid; ELISA: Enzyme-Linked Immunosorbent Assay; IL-6: interleukin-6; GC-MS: gas chromatography-mass spectrometry; HPLC: high performance liquid chromatography; NAD^+^: nicotinamide adenine dinucleotide; NAD(H): total nicotinamide adenine dinucleotide; Nam: nicotinamide; NF-κβ: nuclear factor-κβ; ^1^O_2_: singlet oxygen; PARP: poly(ADP-ribose) polymerase-1; RNS: reactive nitrogen species; ROS: reactive oxygen species; SIRT1: sirtuin 1; TAC: total antioxidant capacity; TNF-α: tumour necrosis factor alpha

## Competing interests

The authors declare that they have no competing interests.

## Authors’ contributions

JG participated in the design and coordination of the study, acquisition and analysis of samples, analysis and interpretation of data and drafted the manuscript. RG conceived of the study, and participated in its design and coordination and helped to both interpret data and draft the manuscript. MG conducted the carotenoid analysis and revised the manuscript. TAM and KDC carried out the F2-isoprostane analysis and revised the manuscript. AB was involved in the analysis and interpretation of data and manuscript revision. All authors read and approved the final manuscript.
